# Unveiling the Shadows: A Rare Encounter With Cardiac Tamponade Following Influenza B in a Young Female

**DOI:** 10.7759/cureus.61876

**Published:** 2024-06-07

**Authors:** Nazish Najeeb, Anastasiia Polianovskaia, Rabia Najeeb, Lal Muhammad, Carlos A Hernandez

**Affiliations:** 1 Internal Medicine, Capital Health Regional Medical Center, Trenton, USA; 2 Internal Medicine, Mayo Hospital, Lahore, PAK; 3 Internal Medicine, United Health Services (UHS) Wilson Medical Center, St. Johnson City, USA; 4 Internal Medicine, Ross University School of Medicine, Bridgetown, BRB

**Keywords:** emergent subxiphoid pericardial window, morbid obesity, sepsis, influenza b, cardiac tamponade, pericardial effusion

## Abstract

Influenza B infection, although primarily recognized for respiratory symptoms, can lead to rare but severe cardiac complications such as pericardial effusion and cardiac tamponade. We present a case of a 33-year-old female with morbid obesity who initially exhibited flu-like symptoms, was subsequently diagnosed with influenza B infection, and was discharged with symptomatic treatment. Despite initial discharge, she returned with worsening weakness, gastrointestinal symptoms, and shortness of breath. Imaging studies confirmed pericardial effusion with early signs of tamponade, necessitating an emergent intervention. The patient underwent subxiphoid pericardial window and fluid removal, followed by colchicine treatment to prevent recurrence. Our case highlights the importance of recognizing and promptly managing rare influenza-related complications, particularly in patients without significant comorbidities. It underscores the value of a proactive approach, utilizing point-of-care ultrasound and echocardiography for early diagnosis and intervention to mitigate mortality and morbidity risks associated with pericarditis and cardiac tamponade secondary to influenza B.

## Introduction

Influenza, a contagious respiratory illness caused by influenza viruses, can manifest with various symptoms and complications, ranging from mild respiratory symptoms to severe systemic involvement [1]. Mild symptoms of influenza include a sudden onset of fever, cough, sore throat, body aches, and fatigue. It is usually treated by alleviating these symptoms. Although influenza usually resolves without intervention in the general population, it can result in higher morbidity and mortality rates among certain high-risk groups [2]. Influenza can lead to several complications, such as pneumonia, myocarditis, and the worsening of pre-existing conditions [3]. We present a case of a 33-year-old female with morbid obesity who initially presented with symptoms suggestive of influenza B infection and subsequently developed complications including pericardial effusion and cardiac tamponade.

## Case presentation

The patient, a 33-year-old female with a significant past medical history of morbid obesity, initially presented to the emergency department (ED) with complaints of generalized malaise, cough, and chest pain that occurred exclusively with coughing. Other symptoms reported included nausea and three episodes of vomiting within the previous 24 hours. The patient denied experiencing headaches, dizziness, shortness of breath, or abdominal pain. Initial vital signs were notable for a body temperature of 37.8°C (oral), a heart rate of 103 beats per minute, a respiratory rate of 18 breaths per minute, a blood pressure of 111/74 mm Hg, and an oxygen saturation of 97% on room air. Physical examination did not reveal any significant abnormalities; heart auscultation was clear of murmurs or gallops with a regular but tachycardic rhythm, and lung auscultation showed no wheezing or crackles with equal bilateral breath sounds. Laboratory testing confirmed a positive polymerase chain reaction (PCR) for influenza B infection. Management during this initial ED visit included administration of ondansetron for nausea, with improvement in tachycardia noted following oral hydration. The patient was subsequently discharged with instructions for symptomatic care at home and a prescription for ondansetron.

Four days post-discharge, the patient re-presented to the ED with generalized weakness, ongoing vomiting, and diarrhea that had persisted since the initial visit. The patient reported cessation of ondansetron sublingual due to increasing nausea and had attempted to maintain hydration with water. The previous diagnosis of influenza B was discussed, and the patient was informed that she was beyond the therapeutic window for oseltamivir, indicating a course of supportive care. Management during this visit included a prescription for Reglan to attempt to control vomiting, advice to increase fluid intake, and instructions to follow up with her primary care provider. The patient was discharged with precautions to return if symptoms worsened.

Three days later, the patient presented for the third time with a new onset of shortness of breath, dizziness, and worsening generalized weakness. Vital signs on this admission revealed hypotension with a mean arterial pressure (MAP) of 61 and tachycardia of 108 beats per minute. Laboratory findings indicated a mild troponin leak (troponin level at 0.692), mild leukocytosis (WBC of 13.98), elevated lactate levels (lactate at 4.5), and a repeat positive respiratory viral panel for influenza B antigen. Urinalysis suggested a urinary tract infection (UTI) with moderately elevated leukocyte esterase, WBC, and RBC counts above 100. The X-ray and CT chest showed bilateral consolidation, an air bronchogram, and mild pleural effusion. The initial evaluation pointed towards sepsis secondary to UTI and pneumonia secondary to influenza B viral infection. The patient’s management plan included the administration of intravenous antibiotics for presumed sepsis, fluids, and pressor support to address hypotension.

**Figure 1:**
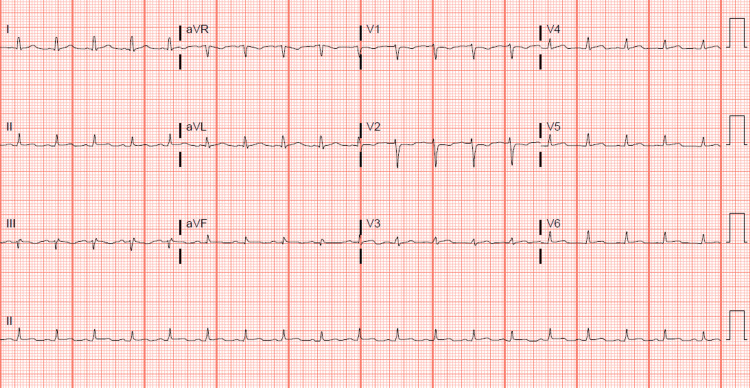
Sinus tachycardia with borderline lateral ST elevation and lower QRS voltage precordial lead.

**Figure 2:**
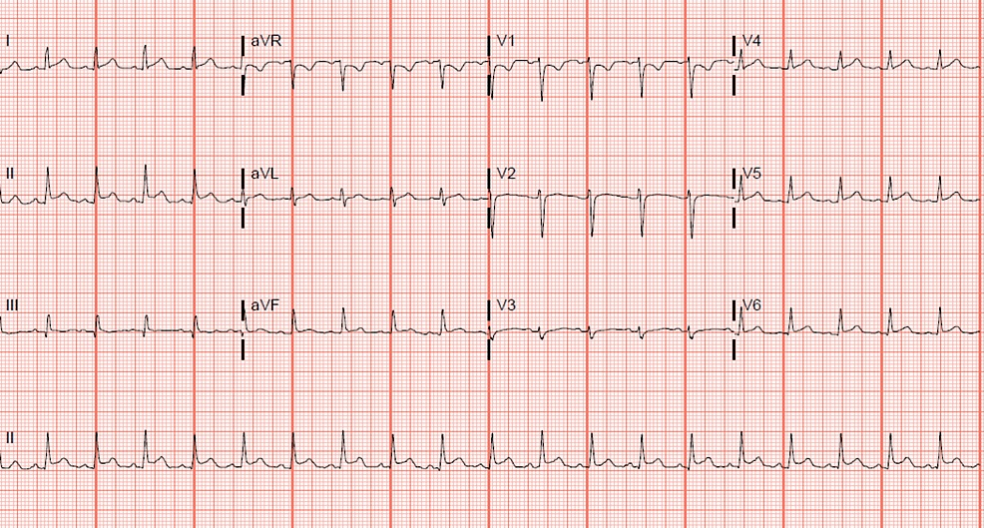
Sinus tachycardia with normal voltage.

The electrocardiogram (EKG) revealed sinus tachycardia with borderline lateral ST elevation and a lower QRS voltage precordial lead (Figure 1) compared to the prior EKG in 2020 (Figure 2).

**Figure 3:**
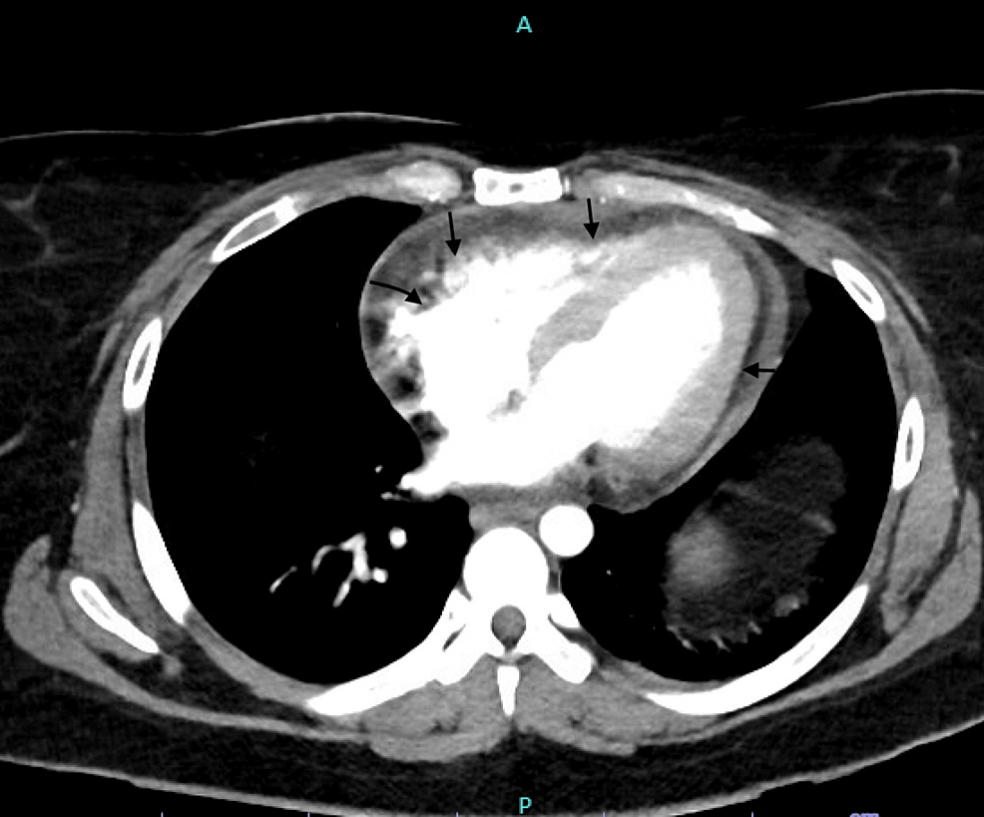
Arrows pointing towards the effusion around the heart.

Imaging studies, including a CT chest showed mild to moderate pericardial effusion (Figure 3).

**Figure 4:**
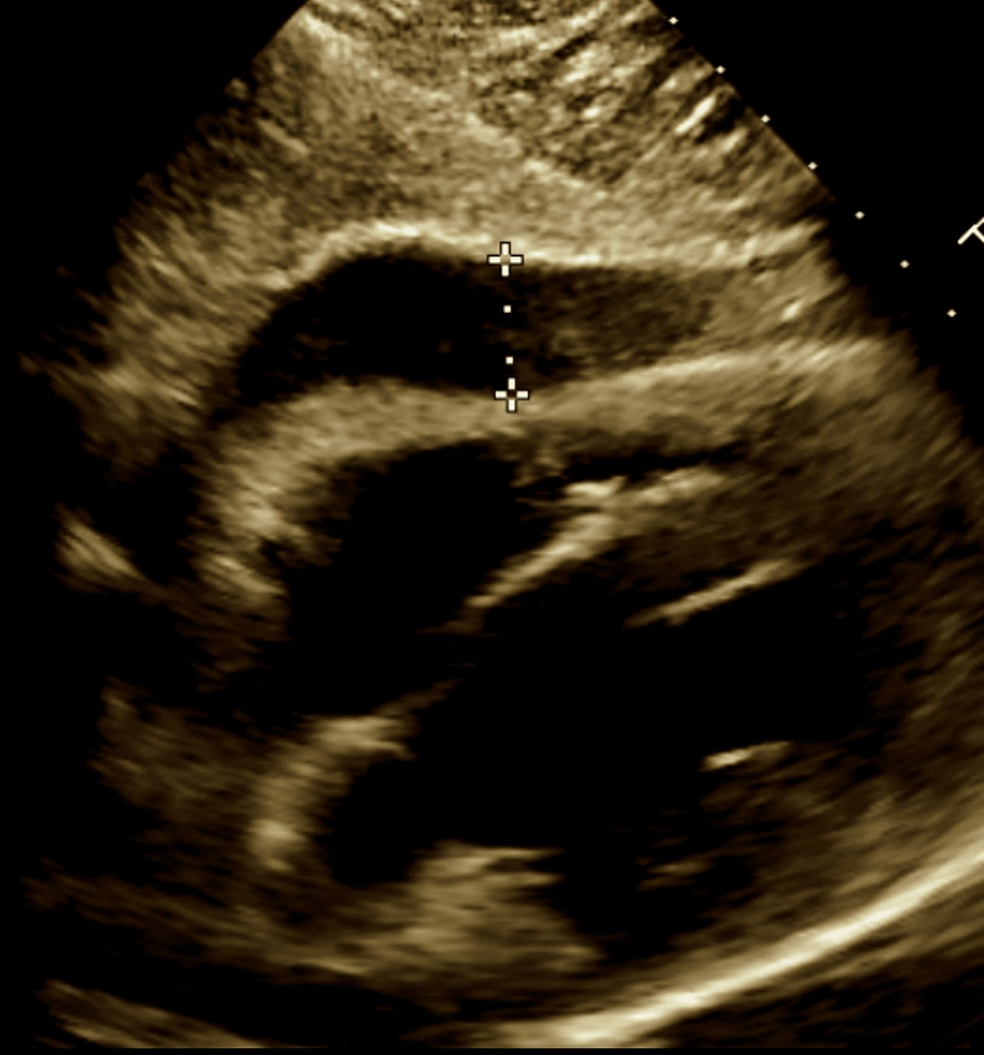
Marked area showing the effusion.

A 2D echocardiogram revealed moderate to large anterior pericardial effusion with early signs of tamponade (Figure 4), EF 45-50%, normal right ventricular function with early diastolic collapse, and a dilated inferior vena cava (IVC).

Given the echocardiographic findings and clinical presentation suggestive of cardiac tamponade, cardiology, thoracic, and cardiovascular surgery were consulted. The patient was evaluated and was emergently taken to the operating room for an emergent subxiphoid pericardial window with drainage of the pericardial effusion. Approximately 300 ml of serous fluid was drained with the restoration of the patient’s blood pressure. The fluid was sent for cytological analysis, culture, and sensitivity testing. Results showed no malignant cells and no growth after three days, including no growth in acid-fast bacillus cultures, respectively. However, white blood cells, debris, and rare mesothelial cells were present.

A segment of the anterior pericardium was sent to pathology, which confirmed benign mesothelial-lined fibrous tissue. A 24-French Blake drain was placed into the anterior space of the pericardium. Within the next five postoperative days, more than 1L of serous or serosanguinous fluid was removed. The drain was removed at the bedside on the sixth postoperative day; it had a minimal output of less than 100 ml a day.

Post-operatively, the patient showed significant improvement, was extubated, and was later transferred from the ICU to a telemetry unit. Follow-up echocardiography showed only trivial pericardial effusion with no evidence of tamponade. The patient was discharged with instructions to complete an antibiotic course for UTI and to continue colchicine for three months.

## Discussion

Influenza, or the flu, is a contagious respiratory illness caused by influenza viruses. It exhibits seasonal patterns, spreading primarily through respiratory droplets and surface contact. The viruses undergo genetic mutations, posing risks, especially to vulnerable groups such as young children and the elderly. Influenza has a significant global impact, causing millions of severe cases and hundreds of thousands of deaths annually [1]. While influenza typically resolves without intervention in the general population, it can lead to elevated morbidity and mortality rates among specific high-risk groups [2]. Influenza can give rise to various complications, including pneumonia, myocarditis, and exacerbations of pre-existing conditions [3].

The influenza virus comprises two primary subtypes: influenza A and influenza B. While both subtypes contribute to epidemic seasonal infections, influenza B is typically regarded as less severe than influenza A and infrequently leads to non-respiratory complications. Influenza B is attributed to <1% of cardiac involvement-related hospitalizations [4]. The heart, responsible for pumping blood, is surrounded by the pericardium, a double-walled sac with a tough fibrous outer layer and a thin serous inner layer [5]. Inflammation of the space between these layers can cause pericarditis, while the accumulation of blood or fluid can result in a pericardial effusion. This buildup can compress the heart, especially the right ventricle, impair its diastolic function, and lead to cardiac tamponade. Diagnostic criteria for cardiac tamponade typically include indications of hemodynamic compromise such as hypotension, pulsus paradoxus, and elevated central venous pressure, coupled with evidence of pericardial effusion observed in imaging studies [6]. Our patient was hypotensive and tachycardic upon presentation. Still, she had vague symptoms of vomiting, diarrhea, generalized weakness, and shortness of breath for approximately one week after she tested positive for influenza B. The initial thought was post-viral pneumonia, considering elevated WBC and shortness of breath leading to septic shock. Respiratory complications are frequently associated with influenza, and the development of post-viral pneumonia is a common occurrence. Our patient presented with right lower lobe infiltrates and slightly elevated procalcitonin levels, leading to antibiotic treatment.

However, the CT scan of the chest revealed mild to moderate pericardial effusion; hence, an echo was ordered and showed moderate to large pericardial effusion with early signs of tamponade, EF 45-50%, normal RV function with early diastolic collapse, and dilated inferior vena cava (IVC). Her clinical presentation and echo findings prompted cardio-thoracic consultation, and she got an emergent subxiphoid pericardial window and the removal of 300 cc of serosanguinous fluid. The fluid was sent for culture and acid-fast bacilli (AFB), which came back negative, and the remainder of the respiratory work, including the respiratory panel, legionella, and strep and methicillin-resistant Staphylococcus aureus (MRSA) screens, was negative as well. The patient reported no personal or family history of autoimmune diseases, rashes, photosensitivity, or other manifestations suggestive of systemic lupus erythematosus (SLE); therefore, the suspicion of autoimmune pericardial effusion was unlikely. A similar case to ours was reported in 2020 in a 32-year-old female with no significant cardiac history who presented four days after testing positive for flu with worsening myalgia and developed significant pericardial effusion while being monitored in the ER in the next 24 hours [7]. Many case reports have been published for post-influenza cardiac tamponade, but there are very limited cases reported with influenza B, as per our knowledge. A review of 25 case reports done in 2023 revealed that early pericardiocentesis has demonstrated efficacy in reducing recurrent pericardial complications, as shown in previous studies. Among the three reported fatalities, associated complications included heart failure, acute respiratory distress syndrome, metabolic acidosis, and asystolic cardiac arrest. Notably, two of the deceased patients did not undergo pericardiocentesis, highlighting the importance of considering this procedure in cases of influenza-related tamponade [8]. Through the creation of a window, we facilitate the removal of fluid from the pericardial sac, thereby providing space for the right heart to expand. This action increases preload and cardiac output, leading to improved hemodynamics. Our patient experienced a gradual reduction in pressor support as a result. The pericardial window was removed after six days, and the subsequent day echo showed minimal resolution of pericardial effusion and was treated with colchicine to prevent a recurrence [6].

Neuraminidase inhibitors and vaccines have been utilized for the prevention and treatment of influenza and its complications. Oseltamivir, when administered orally or enterically, is the recommended antiviral for patients experiencing severe, complicated, or progressive influenza illnesses who are not hospitalized. It is also recommended for hospitalized influenza patients. However, there is insufficient data to advocate for the general use of inhaled zanamivir and intravenous peramivir in patients with severe influenza disease [9]. Our patient was out of the window of 48-72 hours, and oseltamivir was not given.

Our case underscores the importance of considering the rare presentation and complications of influenza B when managing patients without significant comorbidities or cardiac history. It emphasizes the need to avoid bias and skepticism, opting instead for proactive treatment of less common presentations of pericarditis and cardiac tamponade secondary to influenza B, timely diagnosis with point of care ultrasonography (POCUS), bedside echo, and intervention to mitigate mortality and morbidity risks.

## Conclusions

Pericardial effusion secondary to influenza B represents a rare but potentially life-threatening complication. This case emphasizes the need for vigilance in patients presenting with flu-like symptoms and with or without comorbid conditions, the importance of a comprehensive diagnostic workup like POCUS and bedside echo to identify complications early, and the effectiveness of prompt multidisciplinary management in improving patient outcomes.
